# Maternal outcomes of planned mode of delivery for term breech in nulliparous women

**DOI:** 10.1371/journal.pone.0297971

**Published:** 2024-04-03

**Authors:** Malene Mie Caning, Steen Christian Rasmussen, Lone Krebs

**Affiliations:** 1 Department of Gynecology and Obstetrics, University of Copenhagen, Holbaek Hospital, Holbaek, Denmark; 2 Department of Gynecology and Obstetrics, Herlev Hospital, Herlev, Denmark; 3 Department of Gynecology and Obstetrics, University of Copenhagen, Amager Hvidovre Hospital, Hvidovre, Denmark; 4 Department of Clinical Medicine, University of Copenhagen, Copenhagen N, Denmark; Arba Minch University, ETHIOPIA

## Abstract

**Objective:**

To estimate short- and long-term maternal complications in relation to planned mode of term breech delivery in first pregnancy.

**Design:**

Register-based cohort study

**Setting:**

Denmark

**Population:**

Nulliparous women with singleton breech delivery at term between 1991 and 2018 (n = 30,778).

**Methods:**

We used data from the Danish national health registries to identify nulliparous women with singleton breech presentation at term and their subsequent pregnancies. We performed logistic regression to compare the risks of maternal complications by planned mode of delivery. All data were proceeded and statistical analyses were performed in SAS 9.4 (SAS Institute Inc. Cary, NC, USA).

**Main outcome measures:**

Postpartum hemorrhage, operative complications, puerperal infections in first pregnancy and uterine rupture, placenta previa, post-partum hemorrhage, hysterectomy and stillbirth in the subsequent two pregnancies.

**Results:**

We identified 19,187 with planned cesarean and 9,681 with planned vaginal breech delivery of which 2,970 (30.7%) delivered vaginally. Planned cesarean significantly reduced the risk of postoperative infections (2.4% vs 3.9% adjusted odds ratio (aOR): 0.54 95% confidence interval (CI) 0.44–0.66) and surgical organ lesions (0.06% vs 0.1%; (aOR): 0.29 95% CI 0.11–0.76) compared to planned vaginal breech delivery. Planned cesarean delivery in the first pregnancy was associated with a significantly higher risk of uterine rupture in the subsequent pregnancies but not with risk of postpartum hemorrhage, placenta previa, hysterectomy, or stillbirth.

**Conclusion:**

Compared to planned vaginal breech delivery at term, nulliparous women with planned cesarean breech delivery have a significantly reduced risk of postoperative complications but a higher risk of uterine rupture in their subsequent pregnancies.

## Introduction

Breech presentation at term occurs in 3–4% of all singleton pregnancies [1, 2].

Up until the 1960´s women were expected to deliver vaginally regardless presentation. During the 1960´s and 1970´the rate of cesarean for breech increased and during the 1980´s and 1990´s the rate was about 80% in Denmark [3]. After the publication of the results of the Term Breech Trial in 2000 the rate of cesarean at term breech increased and has since been around 90% with small variations between birthplaces in Denmark [4, 5]. Thus, at present, the majority of term breech deliveries are by planned cesarean delivery (CD); in Denmark, approximately 10% are vaginal deliveries (VD) [3, 6].

Planned vaginal breech delivery is associated with a small increased risk of perinatal and neonatal morbidity and mortality. Several descriptive studies and a meta-analysis published after 2015 report a higher relative risk of low Apgar scores, neonatal birth trauma, admission to Neonatal Intensive Care Unit (NICU), and neonatal mortality [7–11] in association with planned vaginal delivery compared to planned cesarean delivery.

In some countries, obstetricians still point out that the neonatal risks can be reduced by following strict criteria regarding which women are suitable for an attempt at vaginal breech delivery as well as a strict awareness of normal progression during vaginal delivery [12–14].

The neonatal risks should be weighed against the risks of maternal complications associated with a cesarean delivery as well as the risk of complications in the woman’s future pregnancies. It is well documented that cesarean delivery increases the risk of maternal short-term complications including postpartum bleeding and wound infections [15, 16]. However, these risks are increased when the cesarean is performed during labour, compared to a planned cesarean delivery [17]. Cesarean delivery entails a risk of uterine rupture, abnormal invasive placenta, placenta previa, and hysterectomy in subsequent pregnancies [15, 18, 19]. Some studies furthermore, indicate an increased risk of antepartum fetal death [20]. Also, a higher rate of subfertility, ectopic pregnancies, and miscarriages has been linked to a history of a previous cesarean [21–23].

However, the high risk of an emergency cesarean during an attempted vaginal breech delivery is important to bear in mind and include when discussing mode of delivery with the pregnant woman and her partner.

The aim of this study was to investigate short- and long-term maternal complications in present and future pregnancies in relation to planned mode of delivery of breech babies at term in the first pregnancy.

## Materials and methods

We conducted a retrospective register-based cohort study using data from the Danish Medical Birth Registry (DMBR) and the Danish National Patient Registry (DNPR).

The DMBR is a population-based registry. It was established in 1968, computerized since 1973. The registry links together the personal ID number of mother, father, and child. The DNPR was established in 1977 and is the key Danish health register. It covers all inpatient, and since 1995 also all outpatient activity, in Danish public and private hospitals or clinics. Information in the DNPR is based on the codes according to the International Statistical Classification of Diseases (ICD-coding) and Related Health Problems Information regarding any procedure is based on the codes according to the Nordic Medico-statistical Committee classification of surgical procedures [24]. Since 2012, blood loss during delivery has been reported to the DMBR in millilitres (mL). All information regarding maternal characteristics and pregnancy and delivery outcomes was retrieved from the DMBR and the DNPR [6].

We retrieved data from all women with singleton pregnancies who delivered their first child in breech presentation at term in Denmark from 1991 to 2018, both years included. Additional inclusion criterion was pregnancies with no congenital malformations. All information regarding maternal characteristics and pregnancy and delivery outcomes was retrieved from the DMBR and the DNPR.

**Fig 1:**
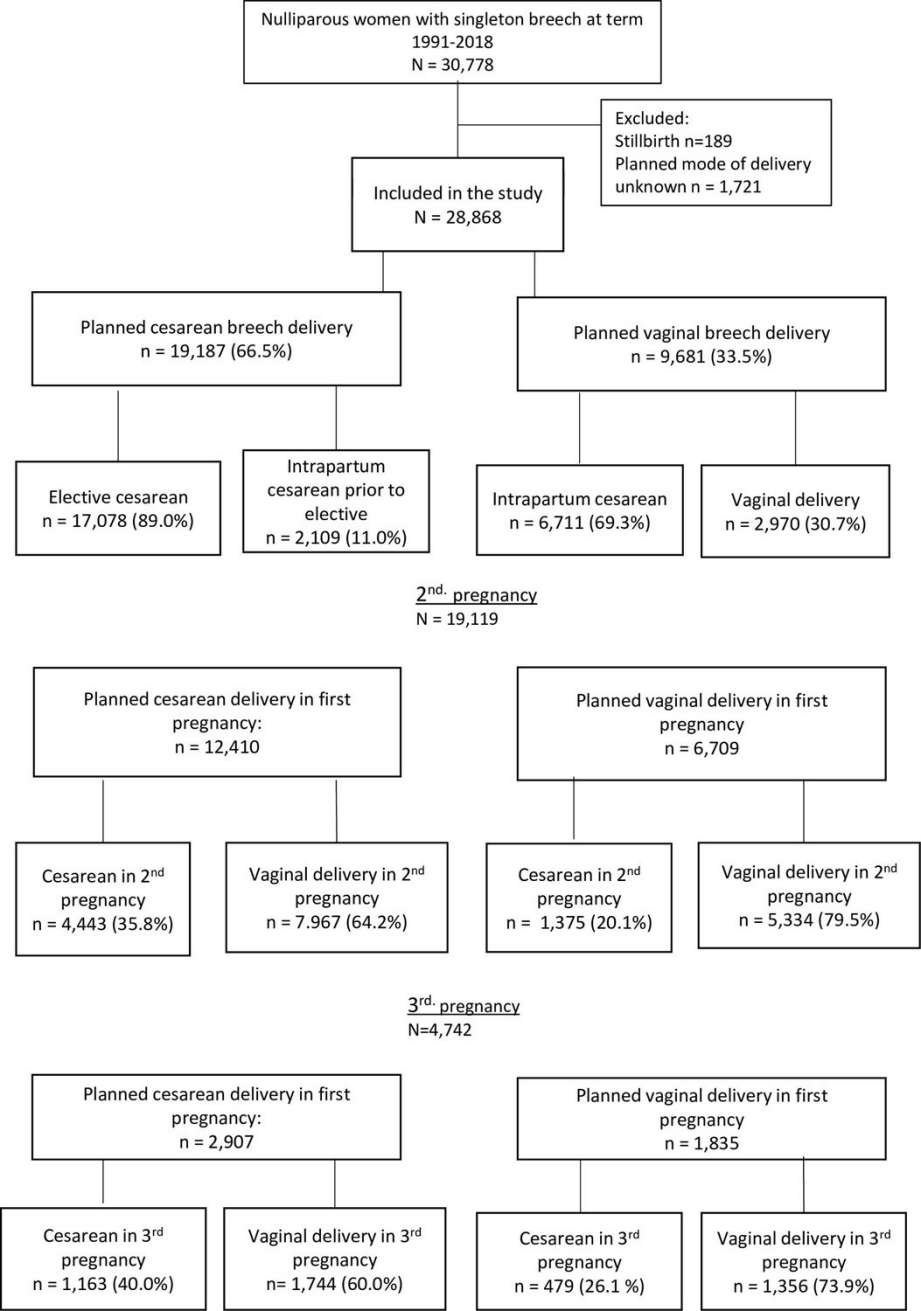
Flow chart of the study population.

Women with stillbirth at first delivery or unknown planned mode of first delivery were excluded (Fig 1). Women who fulfilled the inclusion criteria were identified in the registry, and information on their first and subsequent pregnancies and deliveries was obtained.

The women were categorized according to the planned mode of their first delivery. The definitions of planned mode of delivery were based on the Danish coding system. The four codes for cesarean delivery are (1) emergency CD prelabour, (2) planned (= elective) CD performed prelabour, (3) planned elective CD performed in labour, and (4) emergency CD performed in labour. Planned is in the coding system defined as scheduled >8 hours before the procedure, regardless of whether this was before or during labour. Planned vaginal delivery included all vaginal deliveries and emergency cesarean deliveries in labour (code 4). Planned cesarean delivery included both prelabour and in labour elective cesarean delivery (codes 2 and 3). Due to this design, we were not able to identify women with planned CD who delivered vaginally as they would hence be characterized as planned VD.

Outcome measures related to first delivery were postpartum hemorrhage (a blood loss of 1000 mL or more); infections; surgical organ lesions; re-laparotomy; and postoperative complications defined as a composite outcome including infection, surgical organ lesions (bladder or bowl injuries), or re-laparotomy. In relation to the subsequent pregnancies, the outcome measures included cesarean delivery, placenta previa, uterine rupture, postpartum hemorrhage, hysterectomy, and stillbirth. As data on postpartum hemorrhage before year 2012 are of poor quality, we only included this variable in cases where information on blood loss in mL was available (from 2012). Information on maternal body mass index (BMI) was introduced in the DMBR in 2004.

Supporting information S1 Table includes a detailed list describing codes and specifications for maternal and neonatal morbidity outcomes.

### Statistical analyses

Clinical characteristics and demographic data were reported based on their distribution using counts (percentages) for categorical variables and means (range or standard deviation) for numerical variables.

Outcome measures were compared between cases and controls. Odds ratios (OR) with 95% confidence intervals (95% CI) were calculated by use of marginal two-by-two contingency tables.

Multiple logistic regression was used to adjust for the effect of possible confounding by maternal characteristics, including maternal age, BMI, and smoking. In the regression models any cases with missing values on one or more of the variables were eliminated. The results were expressed as adjusted odds ratios (aOR) with corresponding CIs. All analyses were performed in SAS 9.4 (SAS Institute Inc. Cary, NC, USA).

### Ethical approval

The study was approved by the Danish Data Protection agency (PFI, Region Zealand); REG-209-2016. As a register-based study, no approval from the Danish Research Ethics Committee was obtained, as this was not required according to Danish legislation. For same reason informed consent was not required. All data were fully anonymized upon data analysis.

## Results

We identified a total of 30,778 nulliparous women with a singleton fetus in breech presentation at term during a period from 1991 to 2018. After exclusion of stillbirths (n = 189) and women with unknown planned mode of first delivery (n = 1,721), 28,868 women were included in the analysis and characterized by planned mode of first delivery (Fig 1). A total of 9,681 (33.5%) women had planned vaginal delivery, 2,970 (30.7%) of which delivered vaginally and 6,711 (69.3%) delivered by intrapartum cesarean. Among 19,187 women with planned cesarean delivery, 17,078 (89.0%) delivered by elective cesarean and 2109 (11.0%) by an intrapartum cesarean prior to the planned cesarean delivery. Of the women with planned cesarean delivery in first pregnancy, 12,410 had a second and 2,907 a third pregnancy. In women with planned cesarean in first pregnancy, rates of cesarean in second and third delivery were 35.8% and 40.0%. In women with planned vaginal breech delivery, 6,709 had a second and 1,835 a third delivery. The corresponding cesarean rates were 20.5% and 26.1% (Fig 1).

Maternal characteristics in the first pregnancy by planned mode of delivery are given in Table 1. Compared with women with planned vaginal delivery, women with planned cesarean breech delivery more often had a Body Mass Index (BMI) of 35 or more, were non-smokers, or less than 160 cm in height. The gestational ages (GA) of children born to women with planned cesarean delivery were lower than in children born to women with planned vaginal delivery, and more children were born after 41 gestational weeks in women with planned vaginal delivery.

**Table 1:** Characteristics of the study population (Danish nulliparous women with term breech 1991–2018) by planned mode of delivery in first pregnancy.

Maternal characteristics	Total	Planned Cesarean	Planned Vaginal
	n = 28,868	n = 19,187	n = 9,681
Maternal age (y), mean (SD)	28.7 (4.56)	28.9 (4.64)	28.2 (4.34)
BMI, mean (SD)	23.8 (4.71)	23.9 (4.86)	23.4 (4.22)
BMI >35	483 (1.7)*	410 (3.7)*	73 (1.8)*
n (%)	(n = 15,013)	(n = 10,999)	(n = 4,014)
Maternal height (cm), mean (SD)	168.0 (6.43)	168.1 (6.49)	168.0 (6.25)
Maternal height lower than 160 cm	13,772 (47.7)*	902 (8.2)*	327 (8.1)*
n (%)	(n = 15,096)	(n = 11,065)	(n = 4031)
Smoking, n (%)	5,528 (19.1%)	3,563 (18.6%)	1,965 (20.1)
GA at delivery (days), mean (range)	275.5 (259.9–305.8)	273.75 (260–303.5)	277.25 (259.75–308)
GA at delivery >41 weeks, n (%)	2673 (9.3)	1090 (5.7)	1583 (16.4)
Birth weight (g) mean (SD)	3329.4 (493.7)	3349.0 (484.0)	3290.6 (510.3

*Percentage in women with pre-pregnancy height and weight recorded in the Danish Medical Birth Registry (introduced year 2004)

Y, years; BMI, body mass index; GA, gestational age; d, days; g, grams

Table 2 presents the risk of short-term complications in nulliparous with breech delivery at term by intended mode of delivery.

**Table 2:** Risk of maternal complications in first pregnancy by planned mode of breech delivery (Danish nulliparous with term breech presentation, 1991–2018).

Outcome	Planned Cesarean	Planned Vaginal	OR (95% CI)	*aOR* *
	n = 19,187	n = 9,681		
Intrapartum cesarean n (%)	2,109 (11.0)	6,711 (69.3)	*NA*	*NA*
Postpartum hemorrhage (1000 mL or more) n (%)	242 (5.1)† (n = 4,716)	90 (4.8)† (n = 1,869)	1.06 (0.82–1.36)	1.04 (0.80–1.33)
Infection n (%)	422 (2.2)	333 (3.4)	0.57 (0.46–0.70)	0.55 (0.45–0.68)
Surgical organ lesion n (%)	11 (0.06)	10 (0.10)	0.32 (0.13–0.84)	0.29 (0.11–0.76)
Re-laparotomy n (%)	27 (0.14)	36 (0.37)	0.69 (0.15–3.23)	0.77 (0.16–3.63)
Postoperative complications^§^ n (%)	456 (2.4)	373 (3.9)	0.55 (0.45–0.68)	0.54 (0.44–0.66)

mL, millilitre; OR, odds ratio, CI, confidence interval; aOR, adjusted odds ratio; NA, not applicable

*Adjusted for maternal age, BMI, and smoking

^†^Population in which amount of postpartum bleeding was registered in millilitres (mL) (introduced year 2012).

^§^Composite outcome including infection, surgical organ lesion hysterectomy, or re-laparotomy

Planned vaginal delivery was associated with a significantly higher risk of infection and surgical organ lesions compared to planned cesarean delivery. There was also a reduced risk of re-laparotomy, although this was not statistically significant. Using a composite outcome for postoperative complications (infection, surgical organ lesion and re-laparotomy), the risk of a postoperative complication was significantly reduced in women with planned cesarean delivery (373 (3.9%) versus 456 (2.4%); aOR of 0.54 [CI 0.44–0.66]). There was no statistical difference between planned mode of delivery regarding postpartum hemorrhage (>1000 ml).

Table 3 presents the risk of maternal complications in subsequent pregnancies. The women with planned cesarean breech delivery in the first pregnancy had a higher risk of repeated cesarean delivery in their subsequent pregnancies, 4,443 (35.8%) versus 1,375 (20.5%); aOR 5.3 (CI 4.1–7.1) in second pregnancy and 1,163 (40.0%) versus 479 (26.1%); aOR of 4.5 (CI 3.5–5.8) in third pregnancy. Women with planned cesarean breech delivery in first pregnancy had a significantly higher risk of uterine rupture compared to women with planned vaginal breech delivery, 195 (1.6%) versus 57 (0.9); aOR of 1.85 (CI: 1.37–2.50) in second pregnancy and 20 (0.69%) versus 4 (0.22%); aOR 3.15 (1.08–9.24) in third pregnancy.

**Table 3:** Risk of complications in second and third pregnancy by planned mode of delivery in first pregnancy (Danish nulliparous with term breech presentation, 1991–2018).

**2^nd^ Pregnancy**	**Planned cesarean (first delivery)**	**Planned vaginal (first delivery)**	**OR (95% CI)**	aOR*
**n = 19,119**	**n = 12,410**	**n = 6,709**		
Cesarean delivery, n (%)	4,443 (35.8)	1,375 (20.5)	5.3 (4.0–7.0)	5.3 (4.1–7.1)
Placenta previa, n (%)	173 (1.4)	100 (1.5)	0.90 (0.71–1.12)	0.93 (0.72–1.20)
Uterine rupture, n (%)	195 (1.6)	57 (0.9)	1.85 (1.37–2.50)	1.84 (1.37–2.48)
Hysterectomy, n (%)	9 (0.07)	4 (0.06)	1.21 (0.37–3.94))	1.21 (0.37–3.94)
Postpartum hemorrhage (1000 mL or more) n (%)	304 (7.4)† (n = 3,548)	96 (5.9)† (n = 1,363)	1.25 (0.99–1.60)	1.25 (0.98–1.15)
Stillbirth, n (%)	32 (0.26)	19 (0.28)	0.91 (0.51–1.60)	0.92 (0.52–1.52)
**3**^**rd**^ **Pregnancy**	**Planned cesarean (first delivery)**	**Planned vaginal (first delivery)**		
**n = 4,742**	**n = 2,907**	**n = 1,835**		
Cesarean delivery, n (%)	1,163 (40.0)	479 (26.1)	4.5 (3.5–5.8)	4.5 (3.5–5.8)
Placenta previa, n (%)	25 (0.73)	10 (0.54)	1.68 (0.78–3.62)	1.70 (0.79–3.66)
Uterine rupture, n (%)	20 (0.69)	4 (0.22)	3.15 (1.08–9.24)	3.14 (1.07–9.20)
Hysterectomy, n (%)	4 (0.14)	1 (0.05)	2.51 (0.28–22.51)	2.54 (0.28–22.78)
Postpartum hemorrhage (1000 mL or more), n (%)	47 (4.86) † (n = 968)	19 (4.77) † (n = 398)	1.02 (0.59–1.76)	1.02 (0.59–1.76)
Stillbirth, n (%)	3 (0.10)	8 (0.44)	0.24 (0.06–0.89)	0.24 (0.06–0.90)

mL, millilitre; OR, odds ratio; CI, confidence interval; aOR, adjusted odds ratio

*Adjusted for maternal age, BMI, and smoking

OR = odds ratio, CI = confidence interval, aOR = adjusted odds ratio

^†^Population in which amount of postpartum bleeding was registered in millilitres (mL) (introduced year 2012).

There was no significant association between planned mode of breech delivery and postpartum hemorrhage, placenta previa, and hysterectomy in second or third pregnancies.

Planned vaginal breech delivery was significantly related to stillbirth in third but not in second pregnancy.

Supporting information S2 Table presents a sub analysis for risk of complications based on actual mode of delivery.

## Discussion

### Main findings

In this retrospective register-based cohort study among 28,868 nulliparous Danish women with breech delivery at term, we found that 66.5% intended to deliver by cesarean. Among the women who planned vaginal breech delivery, 69.3% delivered by emergency cesarean. Due to the high risk of secondary cesarean, women with planned vaginal breech delivery were at increased risk of postoperative complications in terms of infections, surgical organ lesions, and re-laparotomy, compared to women with planned cesarean delivery. Having a planned cesarean breech delivery in first pregnancy was associated with a significantly higher risk of uterine rupture in subsequent pregnancies but not to placenta previa, postpartum hemorrhage or hysterectomy. Women with planned vaginal delivery in first pregnancy had a significantly higher risk of stillbirth in their third pregnancy but not in their second. Planned cesarean breech delivery in first pregnancy was associated with a significantly higher risk of repeated cesarean in the two subsequent pregnancies.

### Strength and limitations

This study has the major strength of including a large number of women with information on their complete obstetric history of both intended and actual mode of delivery as well as information on second and third pregnancies and deliveries.

The incidence of emergency cesarean among nulliparous was as high as 69% in the present study, which may be a result of misclassification of categories of cesarean delivery in the register, which of course should be considered as a limitation of the study. In the regression analyses we were able to adjust for some maternal characteristics including maternal age, BMI, and smoking. However, the results may have been affected by other unknown or unmeasured confounders.

Another weakness of the study is that the material is too small to truly evaluate risk of severe and rarely occurring long-term maternal complications such as abnormal invasive placenta and hysterectomies in subsequent pregnancies.

## Interpretation

The present study confirms the findings of a previous study among Danish women showing a very low overall risk of maternal short- and long-term complications regardless of planned mode of breech delivery in first pregnancy [19]. Our data underline the importance of informing women with breech presentation at term about outcomes in relation not only to planned but also to actual mode of delivery and thus take the high risk of an intrapartum cesarean during an attempt of vaginal breech delivery into account. Furthermore, personal counselling should include an individualized assessment of the woman’s chance of having a successful attempt of vaginal breech delivery and include information on short- as well as long-term maternal complications.

What is the best mode of delivery for breech presentation at term has been debated since the 1950s [3]. The vast majority of the analyses are based on descriptive studies. Only a few prospective studies have been conducted. In the Term Breech Trial [4] in 2000, there were no significant differences in short-term maternal mortality or morbidity between groups with planned cesarean delivery and planned vaginal delivery. However, a relatively high percentage (56.7%) of the women randomized to the planned vaginal delivery group actually delivered vaginally and only 36.1% delivered by emergency cesarean.

A French/Belgian observational prospective study (PREMODA) [13] reported a high rate of vaginal deliveries (71%) among women with planned vaginal breech delivery. Unfortunately, this study did not evaluate any maternal outcomes. A secondary analysis of the data from the PREMODA study by Korb et al. [25] focused on short-term severe acute maternal morbidity (maternal death, maternal transfer to intensive care unit, severe postpartum hemorrhage involving blood transfusion, reoperation or pulmonary embolism), and found no differences between the groups with planned cesarean and planned vaginal breech delivery.

An Australian study [8] comparing 10,133 women with term breech presentation used strict criteria for selecting women eligible for vaginal delivery. Of 5,197 women found eligible for vaginal breech delivery, only 352 (6.8%) had planned vaginal delivery. Compared to the group with planned cesarean, the risk of postpartum hemorrhage was higher among the women with planned vaginal delivery (RR 1.69 CI: 1.07–2.68). No difference was found in severe maternal morbidity including cardiac arrest, cerebrovascular hemorrhage, hysterectomy, mechanical ventilation, or post-partum re-admission.

Mattila et al. [26] also compared term breech deliveries according to planned mode of delivery. Of 1,418 term breech deliveries, 406 (28.6%) planned vaginal birth following strict selection criteria. Of these, 338 (83.3%) delivered vaginally. In the group with planned cesarean delivery, 6.5% had postpartum hemorrhage >1000 mL compared to 3.8% in the group with planned vaginal delivery. This difference was not statistically significant. Women in the planned cesarean delivery group more often had puerperal infections including wound infections compared to the women with planned vaginal delivery.

In a Finnish observational study [27] from 2004, 2910 breech deliveries were compared to 133,680 deliveries in cephalic presentation. In the breech group, 56.4% delivered by elective cesarean versus 3.7% in the cephalic group, and 11.4% delivered by emergency cesarean versus 2.8% in the cephalic group. Maternal death occurred only in the cephalic presentation group (3 deaths versus none). Women in the cephalic vaginal delivery group had a higher risk of perineal tears compared to those in the vaginal breech delivery group (OR 0.38 [0.24–0.62]). There were no other differences in maternal morbidity between the groups.

In the secondary analysis of the PREMODA data, Korb et al. [25] also compared vaginal breech deliveries with a control group with cephalic presentation. Not surprisingly, the rate of cesarean was higher in the breech presentation group. The risk of severe acute maternal morbidity was significantly higher in the breech compared to the cephalic presentation group (RR 1.80 [1.02–3.17].

A recently published systematic review and meta-analysis [11] of 32 articles including only studies that focused on the intended mode of breech delivery reports a reduced risk of perinatal morbidity for intended cesarean delivery compared to intended vaginal delivery. The data were sparse on maternal short-term as well as long-term outcomes, hence no conclusions could be drawn.

The present study illustrates the importance of considering the high risk of secondary cesarean during a planned vaginal breech delivery.

Compared to previous studies, we find a high rate of planned vaginal deliveries, thus also a high rate of secondary cesarean. This could be due to an in Denmark cautious approach during attempted vaginal delivery of breech presentation where subacute cesarean is performed if the condition for a successful vaginal delivery is considered poor.

In Denmark, it is recommended that a trial of labour after cesarean be preferred if no other contraindications are present [28]. In this study, 64.2% of the women who gave birth by cesarean in first delivery had a vaginal delivery in their second pregnancy, while 35.8% had a repeated cesarean delivery in their second pregnancy and 40.0% in their third pregnancy. The risk of uterine rupture was twice as high in the second delivery in women with a prior cesarean.

Women with planned vaginal delivery in first pregnancy had a significantly higher risk of stillbirth in their third pregnancy but not in their second. We have no explanation for this finding but based on our results we find it very unlikely that caesarean for breech in first pregnancy is associated to stillbirth in subsequent pregnancies. No other statistical differences in risk of complications in the third pregnancy could be detected.

Women with breech presentation considering planned mode of delivery should be counselled regarding the risk of both neonatal and maternal complications. Overall, planned vaginal as well as planned cesarean breech delivery are safe procedures for the mother and there are only minor differences in maternal the outcomes in the subsequent pregnancies. Future research should evaluate the ability of machine learning models to predict successful vaginal breech delivery and thereby minimize the risk of secondary cesarean in women who wish a vaginal breech delivery.

Also, long-term health effects of planned mode of breech delivery on infant outcomes including auto-immune diseases such as diabetes type-1, asthma and allergies should be further investigated.

## Conclusion

Compared to planned vaginal breech delivery at term, nulliparous women with planned cesarean breech delivery have a significantly reduced risk of postoperative complications in terms of infections and surgical organ lesions but a higher risk of uterine rupture in their subsequent pregnancies.

## Supporting information

S1 Table Detailed list describing codes and specifications for maternal and neonatal morbidity outcomes. (DOCX)

S2 Table Risk of complications in first, second and third pregnancy by actual mode of breech delivery in first pregnancy. (DOCX)

S1 Dataset Dataset Maternal Outcome Breech. (XLSX)
