# Supplementary material for: Maternal outcomes of planned mode of delivery for term breech in nulliparous women
Source: PLoS One. 2024 Apr 3;19(4):e0297971. doi: 10.1371/journal.pone.0297971 (PMC10990212; doi:10.1371/journal.pone.0297971)
Supplement: S1 Table — (DOCX) [file pone.0297971.s001.docx]

**S1 Table:**

Data specification:

Data was derived from the

- National Patient Register (LPR)
- Medical Birth register (MFR)

**Population 1: Mothers.**

The population was formed from the Medical Birth register and constitutes of all women whom in the period 1991-2018 (both years included) gave birth to a singleton child at term in breech presentation.

For the population all access was given from the National patient register to all somatic contacts from public as well as private hospitals.

**Maternal morbidity**

|  | Codes | | |  |
| --- | --- | --- | --- | --- |
| Name | ICD-8 |  | ICD-10 | Specifications |
| Pregnancy, delivery or maternity |  |  | DO* |  |
| Placenta praevia | 651* |  |  |  |
| Uterus rupture | 6599* |  |  |  |
| Complications |  |  | DT814* | Infection after surgery  Incl. postoperative sepsis, abscess, wound infection, pneumonia and urinary tract infection. |
| Cesarean | 63620 |  | KMCA10* |  |
| Hysterectomy | 60940  60960  61020  61100 |  | KMCA33  KLCD00 |  |
| Suture of uterus rupture or bladder | 66120 |  | KMCC00  KKCH00 |  |
| Reoperation | 66800  66820  66840  66860  66890 | opr66800 197701011977010119881231REOPERATION (BLEEDING) 12  opr66800 198901011989010119951231REOPERATION (BLEEDING) 12 100  opr66820 REOPERATION (INFEKTION) 12  opr66820 REOPERATION (INFEKTION) 12 100  opr66840 REOPERATION (S≈RRUPTURE) 12  opr66840 REOPERATION (WOUND RUPTURE) 12 100  opr66860 REOPERATION (WOUND RUPTURE WITH REOPERATION) 12  opr66860 REOPERATION (WOUND RUPTURE WITH REOPERATION) 12 100  opr66890 WOUND REVISION 12  opr66890 WOUND REVISION (OPERATION WOUND) 12  opr66890 WOUND REVISION (OPERATION WOUND) 12 100 | KMWW* |  |
| Treatment of wound on truncus |  |  | KQBB* | Treatment of wound on truncus |
| Hemorrhage | 653* | PARTUS COMPL.EX HAEMORRHAGIA POST PART.CAUS.ATONI.UTERI OA | KMBA*  DO72* | Evacuation of uterus after delivery  Postpartum bleeding.  Hemorrhage registered by the end at a operating- or delivery-room.  Late postpartum bleeding after 24 hours after delivery. |
| Infections | 670*  68219  89800  89720  89620  830* | ENDOMETRITIS PUERPERALIS incl. fever or sepsis in the puerperium.  PHLEGMONE,ABSCESSUS ET LYMPHANGITIS ACUTA TRUNCI  INCISIO ET SUTURA (DELAY) 15  opr89800 197304011973040119881231  EXCISIO VULNERIS CUTIS  SUTURA VULNERIS CUTIS  INCISIO ABSCESSUS CUTIS |  |  |
|  | ** incl.possible sublevels* | | |  |

**Population 2. Children born by mothers in population 1.**

From the Medical Birth Register data was derived from all living born children born at term in breech presentation by mothers in population 1 in the period 1991-2018.

All access was giving from the National patient register to all somatic contacts in public as well as private hospitals.

| Diagnose | ICD-8 | ICD-10 | Specification |
| --- | --- | --- | --- |
| Asthma | 49300  49309 | DJ45* |  |
| Diabetes | 24900  24909  25000  25009 | DE10* |  |
| Inflammatory bowel disease | 56300  56301  56302  56308  56309  56319  56399 | DK50*-51* |  |
| Celiac disease | 26900 | DK900 |  |
| Overweight | 27799 | DE66* |  |
| Juvenile arthritis | 71209  71239  71599 | DM08* |  |
| Cerebral palsy | 34399  34499 | DG80* |  |
| Asphyxia | 776* | DP21* |  |
| Birth lesions | 772* | DP10*-DP15* |  |
| Brain bleeding |  | DP52* |  |
| Cramps in newborn | 780* | DP90* |  |
| Cerebral disturbances in newborn |  | DP91* |  |
| Infections (sepsis in newborn) |  | DP39* |  |
| Ventilator treatment |  | DP22* |  |
|  |  | BMFL38B | Therapeutic hypothermia treatment after perinatal asphyxia |
|  |  | BGFC32*  KFX | CPAP therapy  Special procedures for extra corporal or assisted circulation |
| ** incl.possible sublevels* | | |  |
