# Supplementary material for: Maternal outcomes of planned mode of delivery for term breech in nulliparous women
Source: PLoS One. 2024 Apr 3;19(4):e0297971. doi: 10.1371/journal.pone.0297971 (PMC10990212; doi:10.1371/journal.pone.0297971)
Supplement: S2 Table — (DOCX) [file pone.0297971.s002.docx]

**S2 Table. Risk of complications in first, second and third pregnancy by actual mode of breech delivery in first pregnancy (Danish nulliparous with term breech presentation, 1991-2018)**

| **Outcome in first pregnancy**  **N = 28.868** | **Elective Cesarean**  **n = 17.078** | **Intrapartum cesarean prior to elective cesarean**  **n = 2109** | **Intrapartum cesarean**  **N = 6711** | **Vaginal delivery**  **N = 2970** | **OR (95% CI)**  **Intrapartum cesarean vs. elective cesarean** |
| --- | --- | --- | --- | --- | --- |
| Postpartum hemorrhage^🕇^, n (%) | 216 (1.26) | 25 (1.19) | 65 (0.97) | 26 (0.88) | 0.76 (0.58-1.01) |
| Infection, n (%) | 356 (2.08) | 66 (3.13) | 297 (4.43) | 36 (1.21) | **2.18 (1.86-2.54)** |
| Surgical organ lesion, n (%) | 8 (0.05) | 2 (0.09) | 10 (0.15) | NA | **3.18 (1.26-8.07)** |
| Re-laparotomy, n (%) | 26 (0.15) | 1 (0.05) | 30 (0.45) | 6 (0.2) | **2.95 (1.74-4.94)** |
| Postoperative complications^§^, n (%) | 387 (2.27) | 69 (3.27) | 329 (4.9) | 44 (1.48) | **2.22 (1.91-2.58)** |
| **2^rd^ Pregnancy**  **n = 19.119** | **Elective cesarean (first delivery)**  **n = 11.305** | **Intrapartum cesarean prior to elective cesarean**  **(first delivery)**  **n = 1105** | **Intrapartum cesarean (first delivery)**  **N = 4680** | **Vaginal delivery (first delivery)**  **N = 2029** | **OR (95% CI)**  **Intrapartum cesarean vs. elective cesarean** |
| Placenta previa, n (%) | 166 (1.47) | 7 (0.63) | 77 (1.65) | 23 (1.13) | 1.12 (0.85-1.47) |
| Uterine rupture, n (%) | 178 (1.57) | 15 (1.36) | 53 (1.13) | 6 (0.3) | **0.72 (0.53-0.97)** |
| Hysterectomy, n (%) | 6 (0.05) | 3 (0.27) | 4 (0.09) | 0 | 1.61 (0.45-5.71) |
| Postpartum hemorrhage^🕇^, n (%) | 267 (2.36) | 36 (3.26) | 78 (1.67) | 19 (0.94) | **0.70 (0.54-0.90)** |
| Stillbirth, n (%) | 32 (0.29) | 0 | 14 (0.30) | 5 (0.25) | 1.06 (0.56-1.98) |
|  |  |  |  |  |  |
| **3^rd^ Pregnancy**  **n = 4742** | **Elective cesarean (first delivery)**  **n =2642** | **Intrapartum cesarean prior to elective cesarean**  **(first delivery)**  **n = 265** | **Intrapartum cesarean (first delivery)**  **N = 1231** | **Vaginal delivery (first delivery)**  **N = 604** | **OR (95% CI)**  **Intrapartum cesarean vs. elective cesarean** |
| Placenta previa, n (%) | 24 (0.91) | <5 (0.38) | 7 (0.57) | <5 (0.50) | 0.62 (0.27-1.45) |
| Uterine rupture, n (%) | 15 (0.57) | 5 (1.89) | <5 (0.32) | 0 | 0.57 (0.19-1.72) |
| Hysterectomy, n (%) | <5 (0.15) | 0 | <5 (0.08) | 0 | 0.54 (0.06-4.80) |
| Postpartum hemorrhage^🕇^, n (%) | 42 (1.59) | 9 (3.4) | 19 (1.54) | <5 (0.33) | 0.97 (0.56-1.68) |
| Stillbirth, n (%) | <5 (0.11) | 0 | <5 (0.08) | 0 | 0.72 (0.07-6.88) |

mL, milliliter; OR, odds ratio, CI, confidence interval; RR, Relative Risk; NA, not applicable

^🕇^1000 mL or more. Population in which amount of postpartum bleeding was registered in milliliters (mL) (introduced year 2012).

^§^Composite outcome mL, milliliter; OR, mL, milliliter; odds ratio, CI, confidence interval; RR, Relative Risk; NA, not applicable
